# The Possibilities of Paternalism in Medicine

**Published:** 1912-09

**Authors:** 


### BUFFALO MEDICAL JOURNAL

**A Monthly Review of Medicine and Surgery**

*EDITOR*

A. L. BENEDICT.

All communications, whether of a literary or business nature, books for review and exchanges, should be addressed to the editor, **228 Summer Street, Cor. Elmwood Avenue, Buffalo, N. Y. Subscribers, advertisers and exchanges please revise address.** Please make personal or telephonic calls before 1 p. m.

THE BUFFALO MEDICAL JOURNAL will publish regularly. full transactions of any professional organization in western New York at cost. Personal notices, brief reports of society proceedings, and the like, are always welcome and will be published without charge.

Subscriptions may commence at any time. $2.00 per year in advance.

VOL. LXVIII. SEPTEMBER, 1912 No. 2

*Don't swat the fly, retail; stop its breeding, wholesale.*

### The Possibilities of Paternalism in Medicine.

THE British medical profession is complaining bitterly about the effects of recent legislation in providing medical care as a matter of governmental aid. Yet from an impartial standpoint, it can scarcely be disputed that the poverty, both of a large part of the east end of London and of other cities and even villages of the whole country, require some adequate system of state aid. Jack London's "People of the Abyss," claiming to have been written from an actual sojourn among and sharing of the life of the English poor, is either a gross exaggeration of facts or a justification of almost any scheme of relief, however socialistic.

In the United States, there are those who already see a cloud, bigger than a man's hand, and threatening to extend over the sky. An increase in wages of a hundred per cent. in a life time has failed to give satisfaction. Prices of almost every commodity have risen, perhaps not proportionately with wages but to such a degree as to cause hardship among those not ordinarily considered "the working class" and to lead the latter class to ask what real increase in income has come about and to demand a constantly increasing wage-scale.

Excepting a few abortive attempts in state legislatures, no movement has been made toward legislation which would threaten the welfare of the American medical profession, like that recently passed in England but it requires no excess of pessimism to note similar tendencies. The interest shown in the crusade of the Medical Society of the County of Erie against contract practice, indicates that this is no local issue. The check on the crusade by the State authorities indicates clearly that the crusade cannot be carried on by intra-professional legislation but can suceed only by practically unanimous voluntary professional refusal of services at cheap rates, wholesale. This can be secured, in turn, only by such an equalization of supply of and demand for medical service, as shall make all or at least, the majority of individual practitioiners, independent.

Paternalistic provision of medical aid is naturally and properly accorded to the poor. Not only is the medical profession itself willing to concede this but it has been the worst offender against the principle that charity should not be abused. It has established a precedent, contrary to that applying to practically every other form of charity—lodging, food, fuel, clothing, education, amusement—that medical charity should be at the expense of the medical profession, not of the community at large.

Free medical aid is provided not only for the poor but for soldiers and sailors, including the militia when in actual service and in some additional respects, and the merchant marine at all times that they see fit to apply for it. It is extended to all classes except the very limited one which actually insists on making payment, in regard to vaccination both against small pox and, to some extent, against typhoid and other diseases. The same statement may be made regarding many forms of diagnostic service.

About a quarter of the total population of the United States is frankly foreign, and has brought with it the expectation of free medicial attendance. There is also a tendency to force charity on many other persons, not requiring it, in connection with all sorts of institutions as for the treatment of tuberculosis, insanity, and the care of epileptics, the blind, the mentally feeble and other more or less dependent individuals even though the family of the patient is abundantly able to pay for medical attendance.

A very significant illustration of the general tendency to demand from the medical profession more than its share of free service, is the fact that salaried medical officers are, with the exception of a comparatively few executive heads, paid merely nominal salaries, as compared with the proportionate skill and sacrifice demanded of teachers, firemen, policemen, laborers, male and female, and other employees of the nation, state, county, city, etc.

When we add to the general provision for the poor and dependent class, the numerous forms of wholesale medical aid provided by governmental units for the sake of securing better hygiene and sanitation, the still more numerous forms of providing for soldiers, sailors, policemen, firemen and other employees of the various governmental units, and the similar provision by a large variety of private corporations (mining, manufacturing, operating railroads, steamships, etc.) not to mention benefits of fraternal societies of various kinds and wide extent, it must be acknowledged that the country is criss-crossed with schemes of cooperative medical aid which need only one of three conceivable forces to render them as complete as that with which the British profession is now grappling.

These three forces are: 1. A comparatively slight failure of national self-respect or, rather of mental attitude toward the receipt of cooperative philanthropy; 2. A comparatively slight additional pressure of general poverty; 3. Socialistic legislation, as in England, which would undoubtedly follow and set the official seal upon either of the other forces.

As to the first, it has already been noted that at least a quarter of our population is already inclined to regard medical philanthropy as a natural and proper assistance. The rapid increase of the tipping system, the precedent of accepting education, both in schools, libraries, museums, and lecture courses for adults, the extension of public amusements, the steady influx of immigrants almost inevitably point toward a removal of personal pride as a barrier to the acceptance of free medical service. That the other two forces are growing in strength needs no argument.

The average wage of the medical profession, in terms of the average day's labor or of the purchasing power of the dollar, has declined for the simple reason that the latter two standards have been changing in converse relations, while the standard professional fee has remained almost stationary. Even the large fees of surgeons and specialists are either occassional or, considering the radical nature of the relief conferred, relatively cheap as compared with the smaller, repeated fees of a generation ago for necessarily palliative measures.

How can the medical profession prepare itself for the threatened cataclysm or the more probable gradual increase of economic stress? Only by a systematic, intelligent recognition of the inexorable law of supply and demand, a recognition, selfish in one sense but too broad to consider either personal need or perconal absence of economic pressure, and ultimately unselfish in recognizing the equally inexorable law that efficiency, on a broad scale, must be based on properly paid labor. If the demand for medical services is only equaled by the supply, the profession can meet with equanimity any tendency toward private cooperation to provide insurance against sickness and injury, or toward governmental paternalism. And if, as at present, the supply exceeds the demand, it makes very little difference whether socialistic legislation actually occurs or not, for the underlying economic forces will bring about the same disaster, with mere suffering because more gradually and slowly crushing the individual members of the profession.

